# Long-term survival with multiple metastasectomies for pulmonary osteosarcoma: a case report

**DOI:** 10.1186/s40792-020-01106-2

**Published:** 2021-01-13

**Authors:** Satoshi Takamori, Hiroyuki Oizumi, Jun Suzuki, Katsuyuki Suzuki

**Affiliations:** Department of Surgery II, Faculty of Medicine, Yamagata University, 2-2-2 Iida-Nishi, Yamagata, 990-9585 Japan

**Keywords:** Pulmonary metastasectomy, Osteosarcoma, Multiple surgery, Pregnancy

## Abstract

**Background:**

Repeat pulmonary metastasectomy (PM) considerably improves the prognosis of patients with pulmonary metastases of osteosarcoma. Reports have demonstrated a significantly improved prognosis in patients who have undergone repeat metastasectomy for osteosarcoma; however, there have been no reports with more than six metastasectomies. Herein, we describe the long-term survival of a patient following resection of multiple tumors and other treatments for metastatic osteosarcoma.

**Case presentation:**

A 28-year-old woman underwent extensive resection and postoperative adjuvant chemotherapy for right tibial sarcoma. Over the years, she developed repeated pulmonary metastases. First, 116 metastases were removed from the bilateral lungs. After that, multiple PMs of approximately 250 tumors and other treatments for deep metastatic lesions were performed. The patient died of the underlying disease 24 years after the primary surgery.

**Conclusions:**

This case report demonstrates the long-term survival benefit of a multidisciplinary treatment centered on multiple metastasectomies.

## Background

Pulmonary metastasectomy (PM) has become the standard therapy for various pulmonary metastatic malignancies. It has also been performed for pulmonary metastases of osteosarcoma [1–3]. Reports have demonstrated a significantly improved prognosis in patients who have undergone PMs for osteosarcoma; however, there have been no reports of more than six metastasectomies [2, 3]. This case report describes the long-term survival of a patient who underwent PMs of approximately 250 tumors for metastatic osteosarcoma.

## Case presentation

**Fig. 1:**
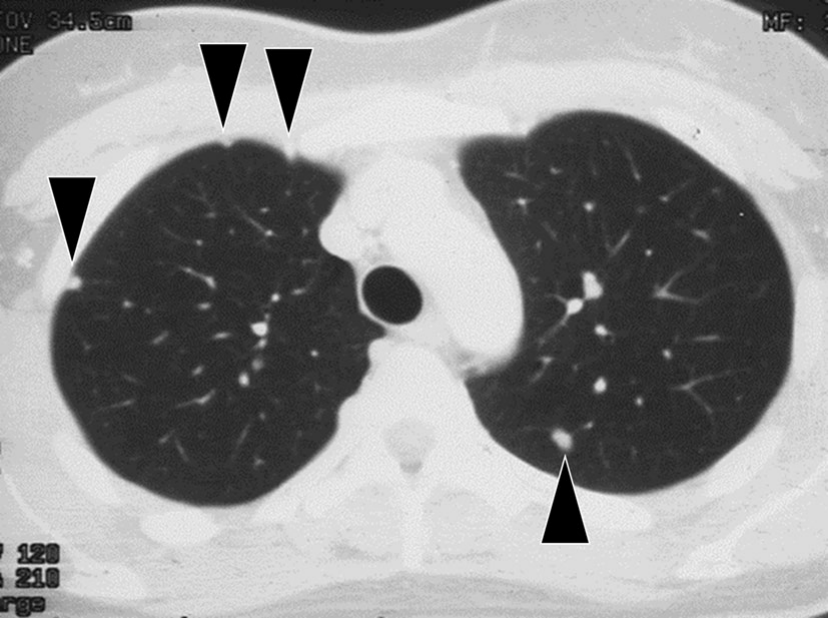
Preoperative image of the first relapse. Computed tomography revealing multiple pulmonary lesions (arrows) (**a**)

**Fig. 2:**
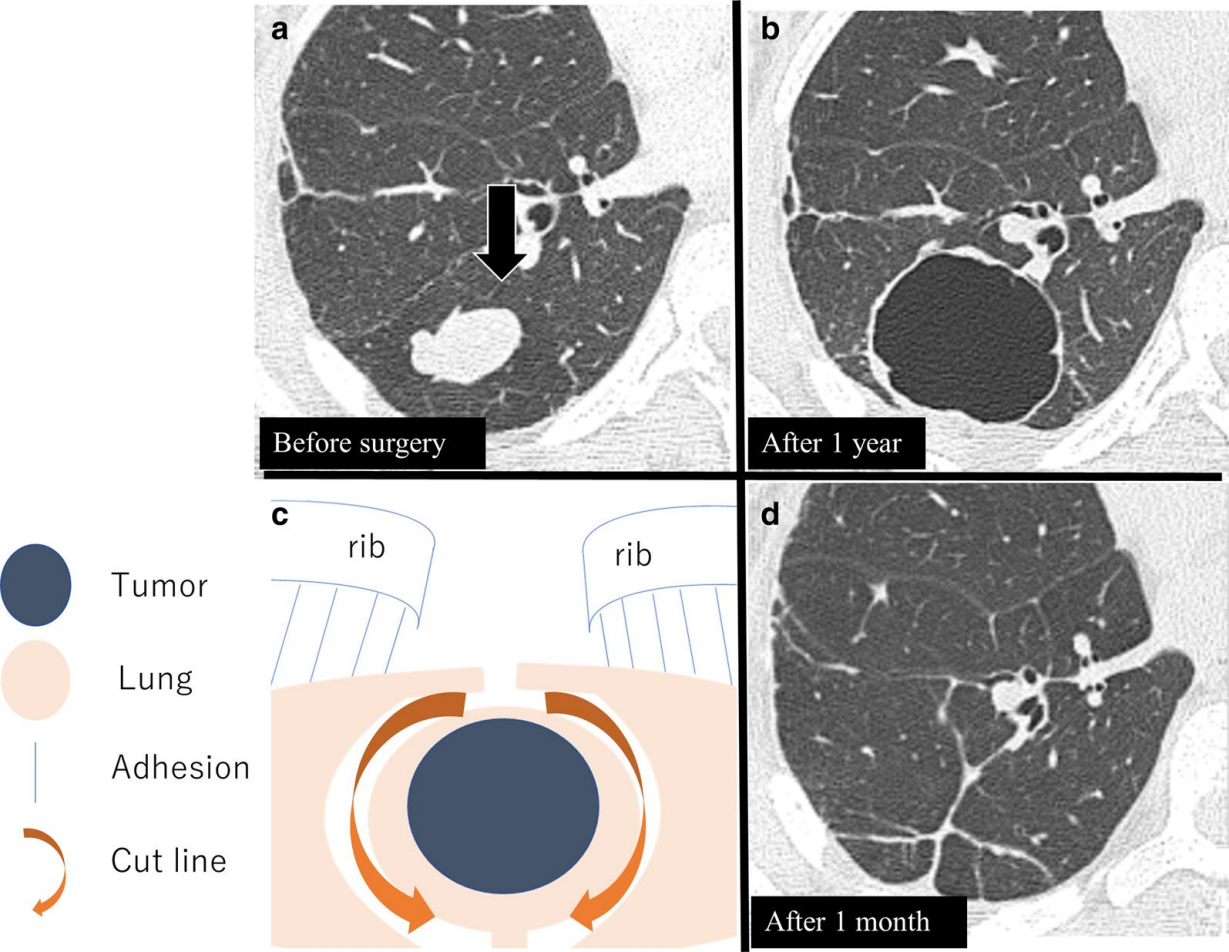
Precision cautery excision method for pulmonary metastasis (X + 17). Preoperative image (**a**). Precision cautery excision method (**b**). Postoperative image (**c**). Postoperative image at 1 month (**d**)

**Fig. 3:**
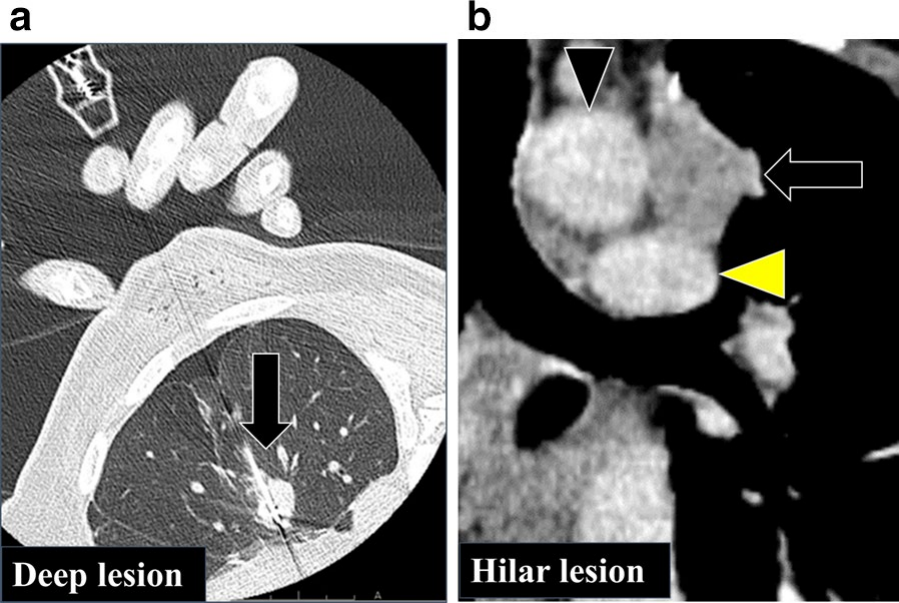
Pretreatment approach for metastatic lesions. Computed tomography revealing a deep pulmonary lesion (arrows), which is treated using radiofrequency ablation (**a**). Radiation therapy is performed for a lesion near the aortic arch (black arrowhead) and pulmonary artery (yellow arrowhead) (**b**)

**Fig. 4:**
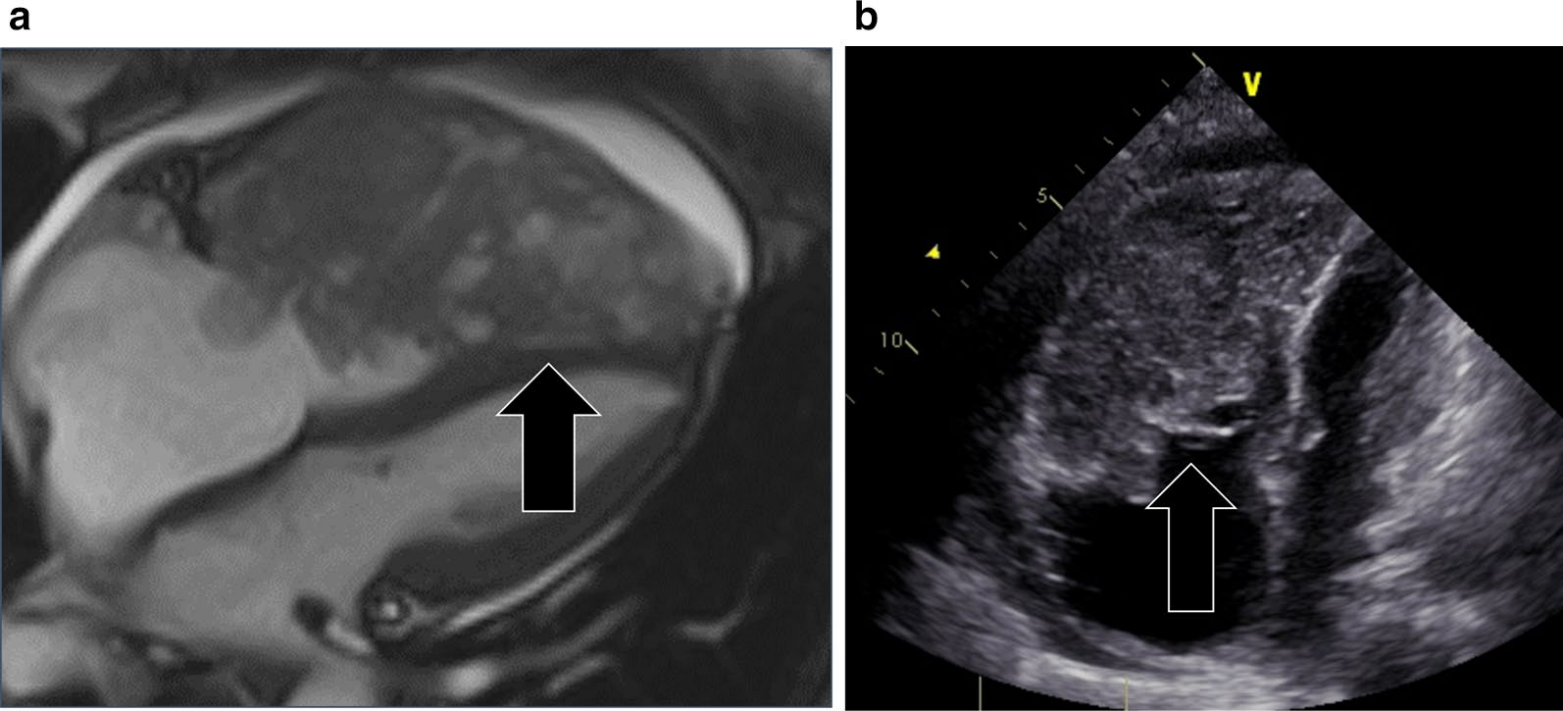
Image at last follow-up. Magnetic resonance imaging (**a**) and echocardiography (**b**) confirm a massive metastasis in the right ventricle (arrow)

The patient was a 28-year-old woman who underwent extensive resection and postoperative adjuvant chemotherapy for right tibial sarcoma. Four years later, multiple bilateral pulmonary metastases were observed (Fig. 1a). The patient refused chemotherapy because she was planning to get pregnant; therefore, she was referred to our division, where she underwent bilateral PMs. We first removed 116 metastases from the bilateral lungs (Table 1). Metastatic tumors were enucleated using neodymium:yttrium–aluminum–garnet (Nd-YAG) laser. Moreover, 3 years after the initial metastasectomy, the patient became pregnant. Multiple pulmonary metastases were observed again 10, 13, 15, 17, and 23 years after the initial surgery. On each occasion, we consulted the attending orthopedic surgeons, who recommended metastasectomy. The patient was informed of the risks of reoperation, but she refused chemotherapy each time; with her consent, metastasectomy was performed at each time point. At 17 years after the initial surgery, a large lung metastasis was observed again (Fig. 2a). Because multiple PMs were performed, extensive and very severe adhesions were found in her chest cavity. As segmentectomy could not be performed, partial resection (precision cautery excision method) was performed. We performed thoracotomy just above the tumor by French window thoracotomy; a Y-shaped incision was made in the pleura. In the precision cautery excision method, after palpating and marking the target tumor, the lung parenchyma was excised in a round shape while securing safe surgical margins. Conventional electrocauterization and vessel sealing system were used to outline the area corresponding to the size of the tumor, and then the lung parenchyma was gradually divided. After the removal of the lung parenchyma, the central side was ligated, and the tumor was removed. The defect was closed using sutures [4] (Fig. 2b, Additional file 1: Video 1). The defect after lung parenchyma resection gradually recovered (Fig. 2c, d). For deep metastases, various treatments were performed. Radiofrequency ablation (RFA) for a deep pulmonary lesion and radiation therapy for a tumor near the aortic arch and pulmonary artery were performed (Fig. 3a, b). The tumors shrank or stopped growing after both treatments. At 50 years of age, the patient underwent her sixth metastasectomy. In total, approximately, 250 tumors were surgically resected during her treatment course (Table 1). A new pulmonary lesion was detected after 1 year, accompanied by a metastatic lesion in the right ventricle (Fig. 4a, b). The patient refused chemotherapy and requested palliative treatment. While on palliative treatment, she died of the underlying disease 24 years after the primary surgery. This case report was approved by the Ethics Committee of the Faculty of Medicine, Yamagata University (#2020-S-41), and informed consent was obtained to publish this report.

**Table 1:** A summary of the patient’s findings during follow-up and the various treatments undertaken

Time from the primary surgery (years)	Approach	Treatment	Device	No. of tumors	Right	Left
X + 4	PLT	Bilateral partial resection^a^	Nd-YAG laser	116	55	61
X + 10	PLT	Bilateral partial resection^a^	Nd-YAG laser	69	18	51
X + 13	PLT	Right S6b subsegmentectomy and partial resection^a^	Electrocautery	55	55	
X + 15	FWT	Left partial resection	Electrocautery	3		3
X + 17	FWT	Right partial resection^a^ Radiofrequency ablation (deep lesion)	Vessel sealing system	1	1	
X + 19		Radiation therapy (hilar lesion) 60 Gy/8 Fr				
X + 23	PLT	Right S9 + 10 segmentectomy and partial resection^a^	Ultrasonic coagulating sears	5	5	

*PLT* posterolateral thoracotomy, *FWT* French window thoracotomy, *Nd-YAG laser* neodymium:yttrium–aluminum-garnet laser

^a^Pulmonary partial resection was performed without stapling

## Discussion

A survey of 146 members of the European Society of Thoracic Surgeons found that 53% of respondents could not place a limit on the number of repeat metastasectomies they would perform [5]. In our case, we opted for repeat PMs because it has been associated with improved survival [2, 3]. Prior studies on PM for osteosarcoma have reported a 5-year overall survival rate of 19–35%, emphasizing the importance of achieving an R0 resection [2, 3]. Moreover, 40% of patients who relapse after pulmonary resection exhibit recurrence in the lung [6]; thus, repeat PM may be a viable treatment option [2, 3].

For patients undergoing repeat PMs, selection for preoperative chemotherapy itself is a negative predictor, irrespective of the response, as only patients with more-aggressive underlying disease processes are selected for multimodal treatment [2]. It has also been suggested that disease progression while undergoing chemotherapy is an independent prognostic factor for poor survival [7]. Our patient was a young woman who wanted to have children. Therefore, chemotherapy was not feasible, and PMs were repeated.

RFA, carbon ion radiotherapy, and radiation therapy have been reported to be effective for unresectable lung lesions and cases in which lung preservation is expected [8–11]. RFA and radiotherapy can be expected to control the tumor [8, 9, 11]. In this case, respiratory function was preserved by performing segmentectomy or partial resection (the precision cautery excision method) [4], while deep metastatic lesions were controlled by RFA and radiation therapy. Pulmonary resection, such as the precision cautery excision method, can minimize pulmonary resection and the decrease in lung function [4]. Even with large tumor size and intrathoracic adhesions, the French window thoracotomy allowed us to perform the operation with minimal impact of adhesions [12]. Repeat metastasectomies were performed using Nd-YAG laser or segmentectomy to preserve the lung function [13, 14]. Hence, the patient could tolerate multiple operations.

In our case, the patient developed right ventricular metastasis. Reports of right ventricular metastasis of osteosarcoma are known, which may be due to hematogenous metastasis of the tumor [15, 16].

## Conclusion

This case report demonstrates the long-term survival benefit of a multidisciplinary treatment centered on multiple metastasectomies. Multiple metastasectomies are risky but can be an option.

## Supplementary Information

**Additional file 1: Video 1.** Precision cautry excision method (X + 17).

## Data Availability

Not applicable.

## Abbreviations

PM: Pulmonary metastasectomy
PLT: Posterolateral thoracotomy
FWT: French window thoracotomy
Nd-YAG laser: Neodymium:yttrium–aluminum–garnet laser
RFA: Radiofrequency ablation
